# Case report: Durable response of immuno-chemotherapy targeting a rare ROS1 fusion-positive extensive-stage SCLC patient after primary resistance to crizotinib

**DOI:** 10.3389/fphar.2025.1522542

**Published:** 2025-04-29

**Authors:** Mengli Qiu, Peiwen Guo, Sisi Wang, Yong Zhu, Siqi Wu, Huiting Peng, Zehuai Guo, Yanmeng Guo, Jieheng Lin, Yang Cao

**Affiliations:** ^1^ The First Clinical School of Guangzhou University of Chinese Medicine, Guangzhou, China; ^2^ The First Affiliated Hospital of Guangzhou University of Chinese Medicine, Guangzhou, China; ^3^ Department of Internal Medicine, Cancer Hospital of Shantou University Medical College, Shantou, China; ^4^ The First Clinical School of Hubei University of Chinese Medicine, Wuhan, China

**Keywords:** SCLC, ROS1, crizotinib, immunotherapy, chemotherapy, primary resistance

## Abstract

**Background:**

Small cell lung cancer (SCLC) is characterized by an exceedingly low mutation rate in oncogenic driver alterations, and there are currently no articles or case reports documenting SCLC patients carrying ROS1 fusions. Tyrosine kinase inhibitors (TKIs) have demonstrated significant efficacy and safety in patients with ROS1 fusion-positive non-small cell lung cancer (NSCLC). However, effective treatment modalities for ROS1 fusion-positive SCLC patients remain poorly defined.

**Materials and Methods:**

We report the first case of an extensive-stage SCLC (ES-SCLC) patient harboring ROS1 fusion, along with TP53, RB1, PTEN, and TERT mutations. The patient exhibited primary resistance to a 3-week course of crizotinib as first-line treatment. Following this, the patient was administered second-line therapy, including chemotherapy coupled with immune checkpoint inhibitor (ICI) and ICI maintenance treatment, resulting in a partial response (PR). Notably, the clinical response to second-line therapy persisted for over 19 months, surpassing the previously reported efficacy of immuno-chemotherapy in ES-SCLC cases (5.7 months) while maintaining a satisfactory quality of life.

**Conclusion:**

We hypothesize that ROS1 fusion may not function as an oncogenic driver alteration in ES-SCLC. Immuno-chemotherapy, not ROS1-TKIs, might provide superior efficacy in ES-SCLC patients with ROS1 fusion.

## Introduction

The ROS proto-oncogene 1 (ROS1) encodes a receptor tyrosine kinase belonging to the insulin receptor family. ROS1 fusions are recognized as actionable oncogenic alterations underlying the carcinogenesis of non-small cell lung cancer (NSCLC), occurring in approximately 1%–2% of NSCLC patients (Drilon et al., 2021). Numerous studies indicate that patients with NSCLC harboring ROS1 fusions exhibit high sensitivity to ROS1 tyrosine kinase inhibitors (ROS-TKIs) (Shaw et al., 2019; Shaw et al., 2017; Malik et al., 2014), leading to their incorporation into clinical guidelines as standard first-line therapies for this patient population. Similarly, ROS1 fusion is extremely rare in patients with small cell lung cancer (SCLC), and there is a notable absence of clinical trials or case reports documenting SCLC patients with ROS1 fusions. For extensive-stage SCLC (ES-SCLC), the standard first-line treatment typically comprises chemotherapy or immunotherapy combined with chemotherapy, yet there is a distinct lack of therapeutic research specifically addressing the subset of ES-SCLC patients harboring ROS1 fusions, which hampers the establishment of clinical evidence for effective treatment strategies.

In this article, we present the first reported case of an ES-SCLC patient harboring a rare TCB1D32-ROS1 fusion, along with TP53, RB1, PTEN, and TERT mutations. This patient exhibited primary resistance to crizotinib after 3 weeks of treatment despite its documented efficacy in ROS1 fusion-positive NSCLC. Following this, second-line treatments, including immune checkpoint inhibitors (ICI) combined with chemotherapy and ICI maintenance, were administered, leading to a partial response (PR) and progression-free survival (PFS) exceeding 19 months so far.

## Case report

**FIGURE 1:**
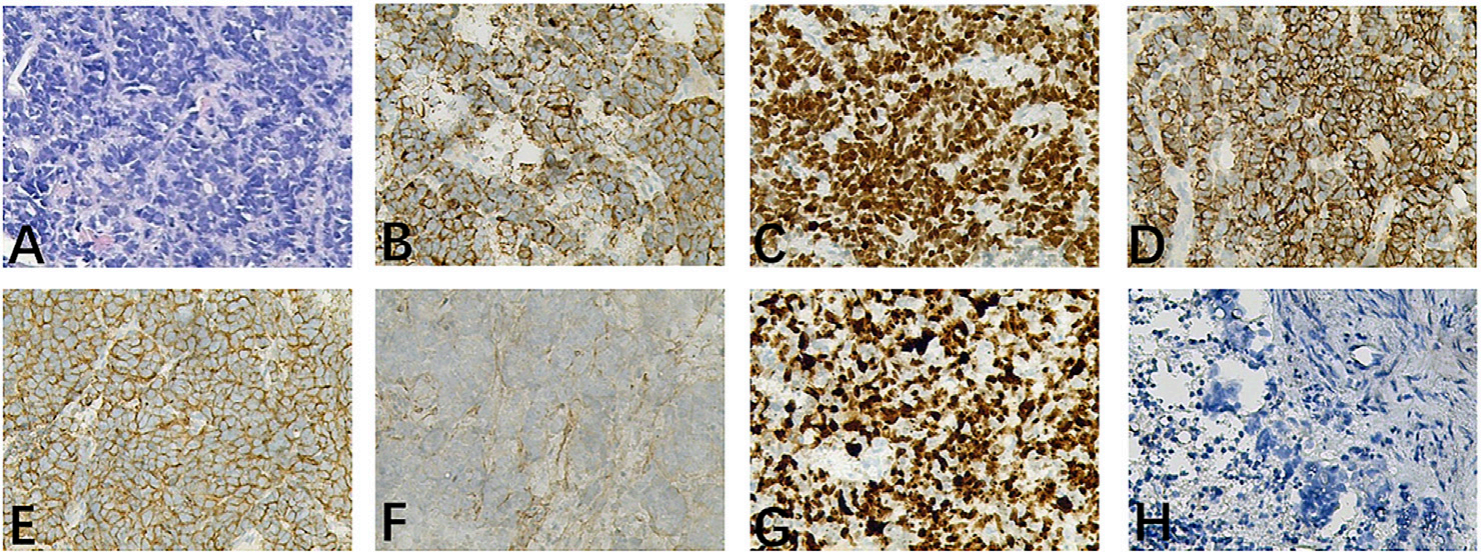
Hematoxylin and eosin staining and immunohistochemistry of tumor issue. **(A)** HE staining suggested that the lesion is consistent with metastatic small cell carcinoma. **(B–G)** The immunohistochemical showed: CK (+), TTF-1 (+), CgA (−), Syn (+), CD56 (+), SSTR-2 (−), Ki-67 (95%+). **(H)** Programmed cell death-ligand 1 staining was negative. HE, hematoxylin-eosin staining; CK, Cytokeratin; TTF-1, Thyroid transcription factor 1; CgA, Chromogranin A; Syn, Synaptophysin; CD56, Neural cell adhesion molecule; SSTR-2, Somatostatin Receptor 2; Ki-67, Ki-67 antigen; TP53, Tumor Protein 53; RB1, RB transcriptional corepressor 1; TERT, Telomerase Reverse Transcriptase; ROS1, ROS proto-oncogene 1, receptor tyrosine kinase.

**FIGURE 2:**
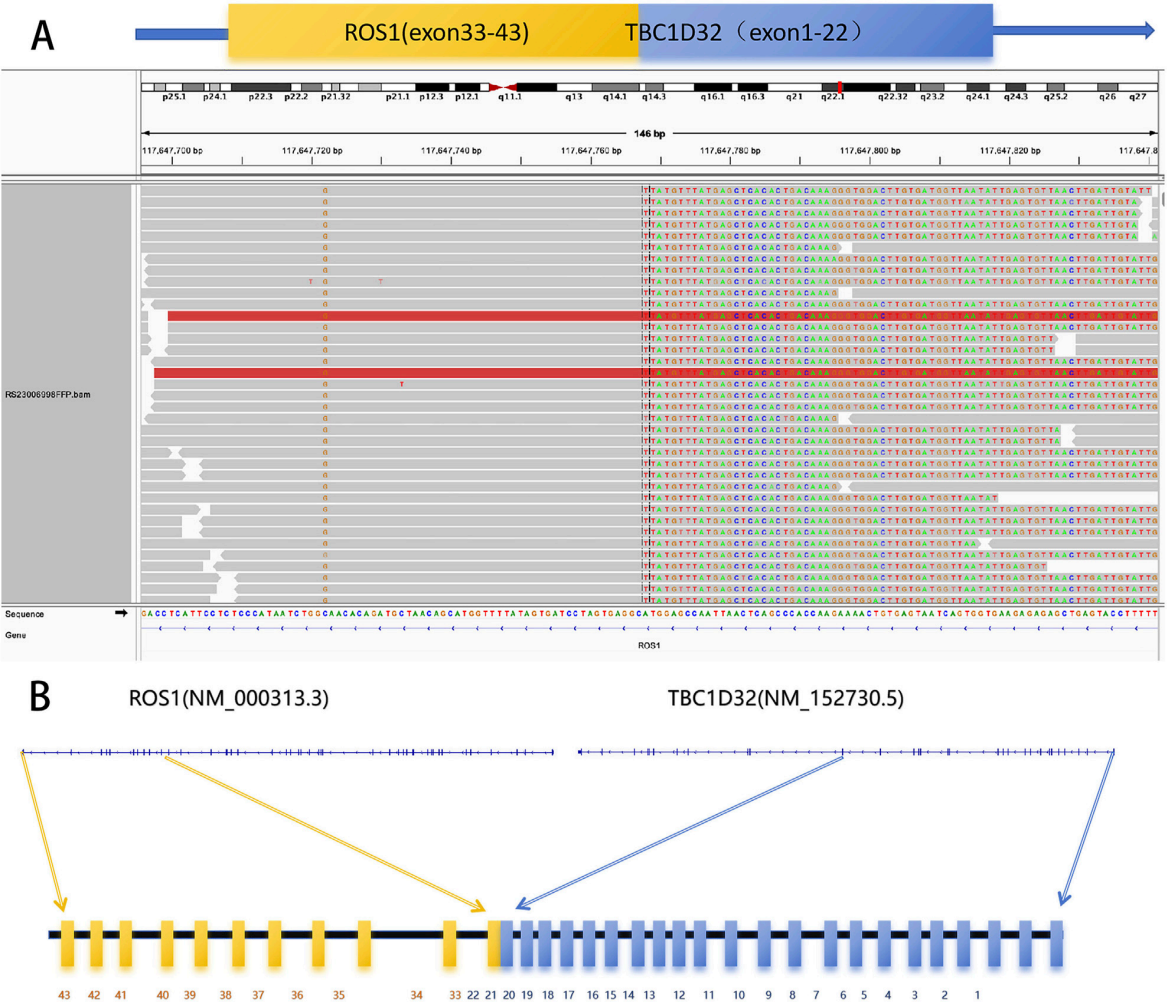
Identification of TBC1D32-ROS1 Fusion in Tumor Samples. **(A)** Integrative Genomics Viewer (IGV) screenshots demonstrate chimeric reads identified by next-generation sequencing (NGS), indicating the presence of the TBC1D32-ROS1 fusion (T22:R33). This fusion arose from a deletion on chromosome 6 [del (6) (q21q22.32)] between the 5′region of TBC1D32 (NM_152730.5) and the 3′region of ROS1 (NM_000313.3), resulting in the fusion of TBC1D32 exons 1-22 to ROS1 exons 33-43. The breakpoint is located at 6:121494094_6:117647767. The gene structure, depicting exons (colored boxes) and introns (gray lines), is shown for TBC1D32 (blue) and ROS1 (yellow). **(B)** Schematic representation of the TBC1D32-ROS1 fusion gene.

A 70-year-old female patient was admitted to the First Affiliated Hospital of Guangzhou University of Chinese Medicine due to a persistent dry cough and breathlessness lasting 1 month. She had a 20-year history of hypertension, well-controlled with regular administration of amlodipine besylate. She had no history of smoking or family history of cancer. A PET scan conducted in January 2023 showed enlarged lymph nodes (LN) in the right supraclavicular and infraclavicular fossa, mediastinal areas, and the left hilum of lung, with increased fluorodeoxyglucose uptake levels, indicating tumor metastasis (Figure 2A). Biopsy and immunohistochemical examination of the right cervical LN confirmed the diagnosis of metastatic small-cell carcinoma (Figure 1). Serum biomarkers, including neuronal specific enolase (NSE) and pro-gastrin-releasing peptide (ProGRP), were elevated at 238.7 ng/mL and 420.3 pg/mL, respectively. A comprehensive review of the patient’s medical history, pathology, and imaging data led to a diagnosis of ES-SCLC per the Veterans Administration Lung Study Group criteria (Micke et al., 2002). Next-generation sequencing (NGS) of tumor tissue specimens from cervical LN identified ROS1 fusion, along with TP53, RB1, PTEN, and TERT mutations; microsatellite stability; tumor mutational burden (TMB) of 34.9 mutations/Mb; PD-L1 (Tumor proportion score: 0%) (As shown in Table 1; Figure 2).

**TABLE 1:** Gene mutations associated with tumor targeted therapy.

Gene	Base changes	Amino acid change	VAF (%)/CN
TP53	Nonsense mutation in exon 6:c.586C>T	p.Arg196^*^	83.80
RB1	Copy number deletions in exons 4-exon6	—	0.6
PTEN	Missense mutation in exon 5: c.275A>G	p.Asp92Gly	83.04
TERT	Promoter mutation: c.219-C>A	—	54.72
ROS1	TBC1D32-ROS1 fusion	—	42.44

TP53, Tumor Protein 53; RB1, RB, transcriptional corepressor 1; TERT, telomerase reverse transcriptase; ROS1, ROS, proto-oncogene 1, receptor tyrosine kinase; VAF, variant allele frequency; CN, copy number.

**FIGURE 3:**
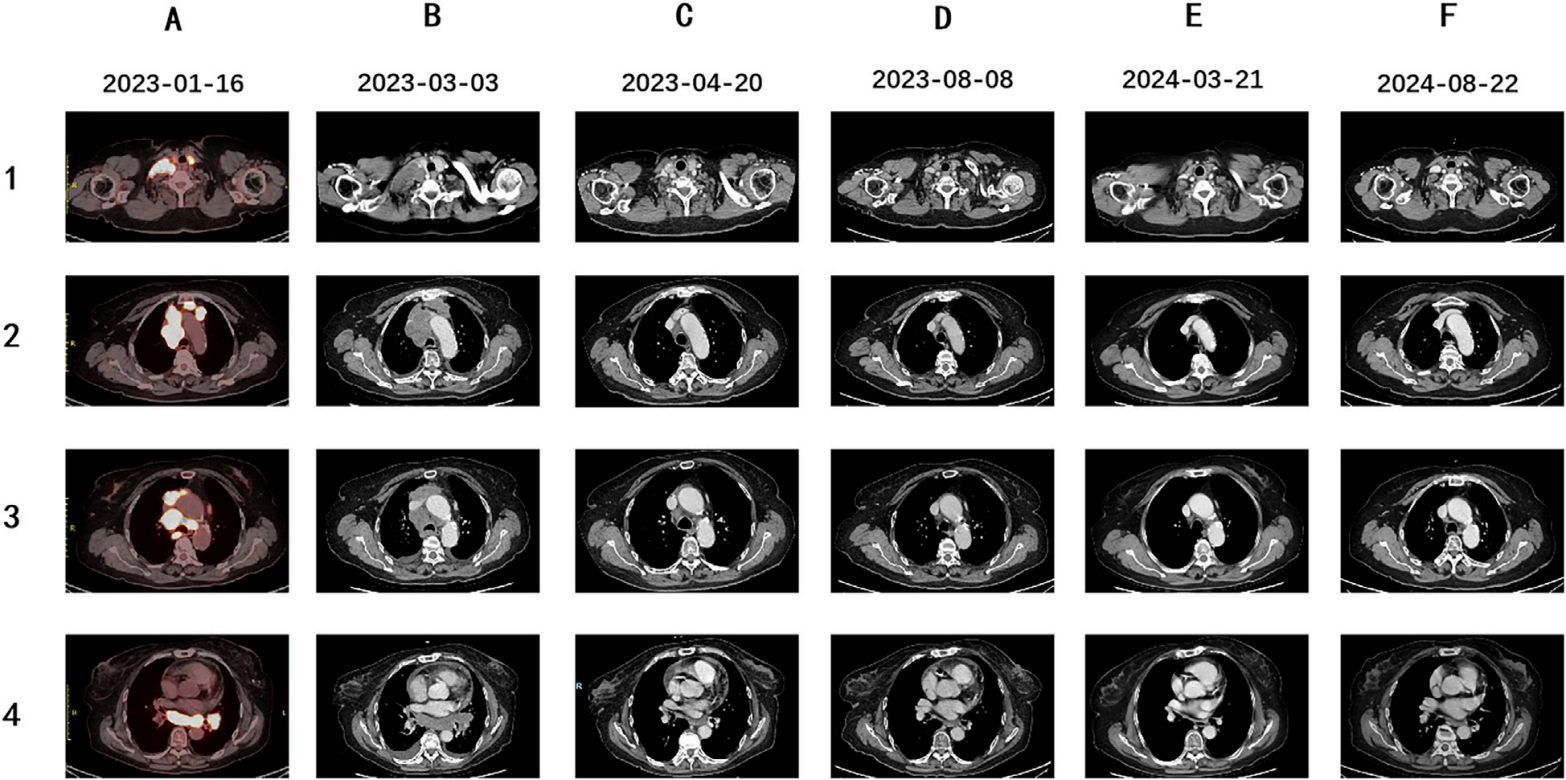
PET-CT and follow-up Enhanced CT images of this patient. **(A)** The patient was diagnosed as ES-SCLC. Pre-treatment PET-CT scan suggested enlarged lymph nodes (LNs) in the right supraclavicular and infraclavicular fossa, mediastinal areas, and left hilum of the lung, with increased fluorodeoxyglucose uptake level, indicating tumor metastasis. **(B)** Enhanced CT scan suggested enlarged area of the initial metastatic lesions in the right supraclavicular and infraclavicular fossa, mediastinal areas, and left hilum of the lung after 3 weeks of crizotinib treatment. **(C)** Enhanced CT evaluation revealed the significant shrinkage of multiple LNs after two courses of Etoposide, Cisplatin and Sapolizumab treatment. **(D–F)** During the therapeutic period of Sapolizumab as maintenance therapy, regular enhanced CT scans suggested stable disease.

**FIGURE 4:**
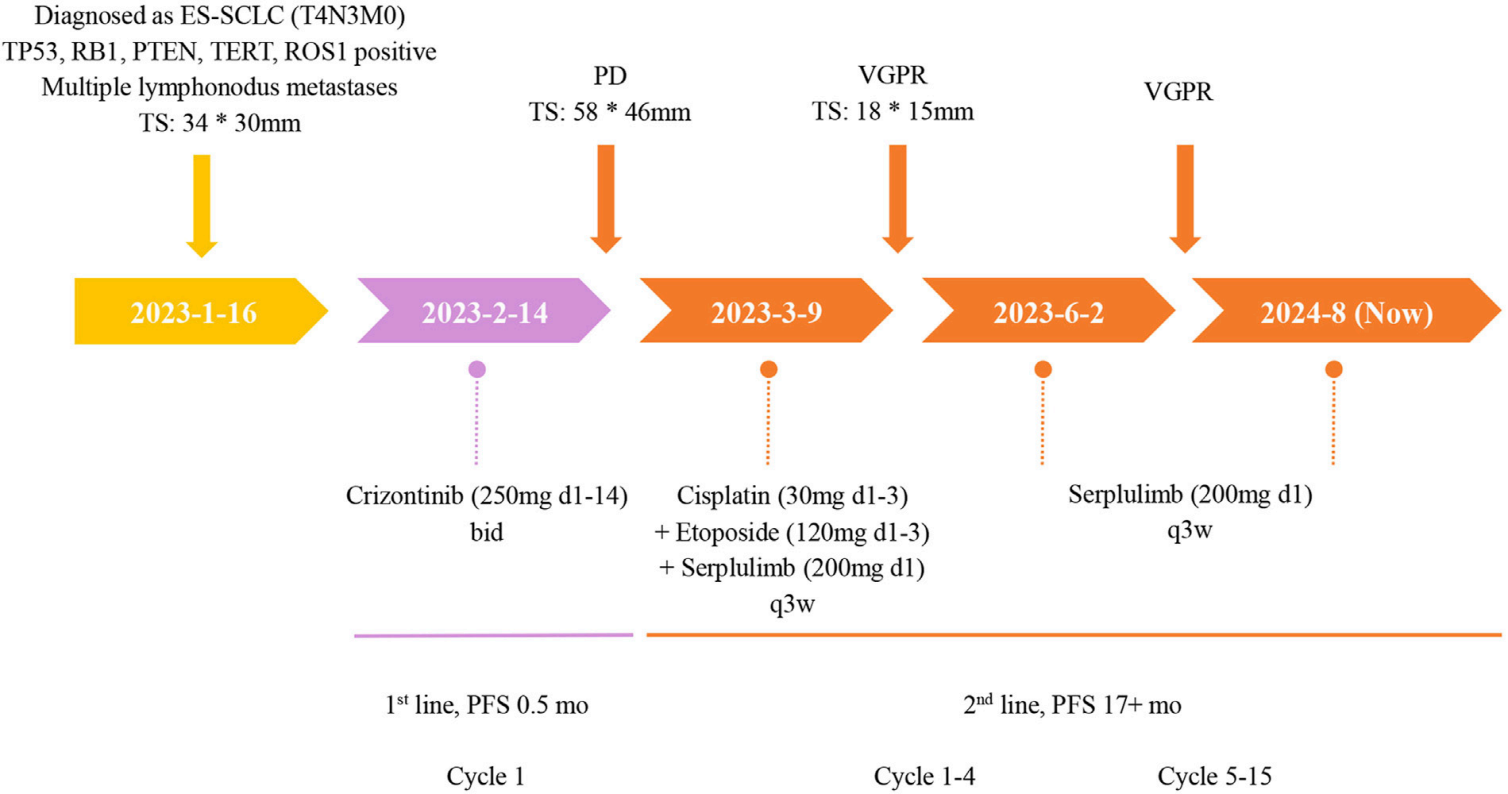
The treatment timeline of this ES-SCLC patient with ROS1 mutation. TS, tumor size; PD, progressive disease; PR, partial response; BID, twice a day; Q3W, once every 3 weeks; wks, weeks; mo, month.

The patient initiated first-line treatment with crizotinib on 14 February 2023, at the standard oral dosage of 250 mg twice daily. However, as treatment progressed, her cough exacerbated, and a subsequent CT scan performed 3 weeks later showed progressive disease (PD). According to the Response Evaluation Criteria in Solid Tumors 1.1 (RECIST 1.1), the CT image disclosed rapid enlargement of multiple lymph nodes throughout the body (Figure 3B). Consequently, on 9 March 2023, the patient commenced a second-line treatment regimen consisting of the ICIs Serplulimab, in combination with etoposide and cisplatin, encompassing four sessions. After two treatment cycles, the CT scan (Figure 3C) demonstrated a PR, and the patient reported a partial alleviation of cough symptoms. On 2 June 2023, the patient transitioned to ICI maintenance therapy with Serplulimab. As of this writing, she has completed over 24 sessions of Serplulimab monotherapy. Her symptoms were relieved, and she displayed a strong willingness to continue treatment. The most recent evaluation in August 2024 showed complete resolution of lung tumor on CT (Figure 3F) with full remission of cough symptoms (NRS [Numerical Rating Scale] score reduced from eight to 0). Detailed assessments of the patient’s CT images, treatment timeline, and serum NSE and ProGRP levels are illustrated in Figures 3, 4, Supplementary Figure S1, and Supplementary Videos S1-6, respectively. All procedures performed in this study were in accordance with the ethical standards of the institutional and/or national research committee(s) and with the Declaration of Helsinki. Written informed consent was obtained from the patient for publication of this case report and accompanying images.

## Discussion

In this article, we report the first case of ROS1 fusion-positive ES-SCLC patient. This patient experienced primary resistance after receiving 3 weeks of oral crizotinib as first-line treatment. Subsequently, the patient received second-line therapy, which included chemotherapy combined with ICI and ICI maintenance treatment, resulting in a PR and PFS exceeding 19 months to date.

ROS1 alterations can be classified into several categories: fusion, overexpression, splice variant, mutation, and amplification (Drilon et al., 2021). As a proto-oncogene located on chromosome 6q22.1, ROS1 encodes a receptor tyrosine kinase. ROS1 fusion prompts the expression of constitutively activated fusion kinases, thereby exerting a potent oncogenic effect (Davies et al., 2012). Similarly, the TBC1D32 gene, also known as C6orf170, encodes a protein associated with ciliary function and is predicted to contain Tre-2, Bub2, and Cdc16 domains, which directly interact with cell cycle-related kinases (Peng et al., 2019). In this case, the targeted NGS found a novel TBC1D32-ROS1 fusion. This variant derives from the fusion of exon 22 of the TBC1D32 gene with exon 33 of ROS1 gene (T22: R33) and preserves the structural domain of ROS1 kinase.

Clinical evidence (Shaw et al., 2014; Shaw et al., 2019) confirms that ROS1 fusion-positive NSCLC patients treated with crizotinib achieve an objective response rate (ORR) of 72%, with median progression-free survival (mPFS) of 19.3 months and median overall survival (mOS) of 51.4 months, demonstrating favorable safety profiles (incidence of grade ≥3 adverse events <10%). Supported by multicenter retrospective analyses and prospective trials, crizotinib has received regulatory approvals from the U.S. Food and Drug Administration (FDA), European Medicines Agency (EMA), and China’s National Medical Products Administration (NMPA) as first-line standard therapy for advanced ROS1 fusion-positive NSCLC. This therapeutic paradigm informed our selection of crizotinib as the primary intervention for the index case. Crizotinib is a multi-target kinase inhibitor with differential inhibitory potency against ROS1, Anaplastic Lymphoma Kinase (ALK), and Mesenchymal-Epithelial Transition (MET). Cell-based assays reveal its IC_50_ (Half maximal inhibitory concentration) values for MET and ALK to be 11 and 24 nmol/L, respectively, while the IC_50_ value for ROS1 ranges from 3.9 to 5.4 nmol/L. Notably, the Ki (Ki Inhibition Constant) value in ROS1 fusion-positive cell lines is 0.6 nmol/L, indicating stronger binding affinity and inhibitory activity toward ROS1 compared to other targets (Zhong et al., 2025). However, contrary to expectations, disease progression occurred in this patient after 3 weeks of treatment.

While crizotinib demonstrates robust efficacy in ROS1 fusion-positive NSCLC, our ES-SCLC case exhibited primary resistance. We propose three potential resistance mechanisms supported by molecular profiling and prior evidence. First, the TBC1D32 fusion partner may induce conformational alterations in the ROS1 kinase domain (e.g., steric hindrance or allosteric effect), thereby interfering with crizotinib binding to the target (Zhao et al., 2024). The breakpoint location and fusion partner identity in ROS1 fusions can influence the efficacy of first-line crizotinib therapy. Li et al. (2024) demonstrated that patients with long fusion variants (exon 32 breakpoints) had significantly shorter mPFS compared to those with short fusions (exons 34/35 breakpoints). Studies (Li et al., 2018) focusing on the CD74 fusion partner have shown that patients with non-CD74-ROS1 fusions had longer PFS (17.63 vs. 12.63 months) and higher ORR (94.11% vs. 73.68%) when treated with crizotinib. Second, SCLC is characterized by high genomic instability and complex signaling networks (Peifer et al., 2012), with core drivers of its development including classic events such as TP53/RB1 co-inactivation and MYC family amplification. In this context, the ROS1 fusion may be present as a “passenger mutation,” and the SCLC may harbor other more critical driver genes or bypass signaling pathways (Liu et al., 2024; Chatterjee et al., 2018; Stockhammer et al., 2024). Finally, the multiple co-occurring gene mutations in this case may synergistically promote tumor progression and induce crizotinib resistance. Studies (Zhang et al., 2021) have shown that ROS1 fusion-positive NSCLC patients with co-occurring oncogenic drivers (e.g., EGFR, MET amplification, or KRAS mutations) or tumor suppressor gene mutations (e.g., TP53, RB1, or PTEN) have significantly shorter PFS compared to those without concomitant mutations. Based on the above evidence, the observed crizotinib resistance in this case may be related to the following factors: ROS1 kinase conformational changes induced by the rare TBC1D32 fusion partner; the unique biological role of ROS1 fusions in SCLC; and the impact of multiple co-occurring gene mutations.

Due to the unsatisfactory outcomes with first-line therapy, this patient received immuno-chemotherapy, followed by immunotherapy maintenance as second-line treatment. This approach aligns with the standard first-line treatment recommended by guidelines for SCLC patients. However, no clinical studies confirmed whether ES-SCLC patients with ROS1 fusions can benefit from ICIs. In this case, the patient achieved over 19 months of PFS following second-line immuno-chemotherapy and immunotherapy maintenance, demonstrating a favorable safety profile. Notably, previous studies suggested that mPFS for ES-SCLC patients receiving first-line Serplulimab treatment coupled with chemotherapy is 5.7 months (Cheng et al., 2022). We hypothesize that this excellent efficacy to immuno-chemotherapy may be related to the presence of multiple gene mutations, including ROS1, TP53, and TERT, as well as a higher TMB (TMB level of this patient was 34.9 mutations/Mb). Biomarker levels, including PD-L1, microsatellite instability (MSI), and TMB, significantly influence the efficacy of immunotherapy in cancer patients (Wang et al., 2021). Li et al. identified a correlation between ROS1 gene alterations and increased TMB levels through analyzing an immunotherapy database, a finding that aligns with our observations, suggesting that ROS1 may serve as a favorable prognostic biomarker for various cancer patients undergoing ICIs treatment (Li et al., 2020). Additionally, Dong et al., investigated multiple lung cancer and immunotherapy databases and found that TP53 mutations could enhance the expression of immune checkpoints and TMB levels. Further clinical trials confirmed the favorable clinical response of ICIs in patients with TP53 mutations (Dong et al., 2017). Moreover, Jiang et al. found a correlation between TERT mutations and increased tumor immunogenicity and antitumor immune inflammation, with cancer patients harboring TERT mutations exhibiting significantly improved OS after receiving ICIs. The predictive value of TERT alterations was independent of tumor mutational burden and microsatellite status (Jiang et al., 2020). This suggests that TERT mutations could function as a potential pan-cancer predictive biomarker for ICI therapy. Considering the above findings and mechanistic analyses, we speculate that the prolonged PFS observed in this patient (currently exceeding 19 months) following immuno-chemotherapy may be attributed to the presence of ROS1, TP53, and TERT mutations, along with a higher TMB level. Moreover, Liu et al. (2019) have shown that crizotinib, in combination with cisplatin, can induce immunogenic cell death in NSCLC, as evidenced by surface exposure of calreticulin and extracellular release of ATP. These damage-associated molecular patterns facilitate the recruitment of dendritic cells into the tumor microenvironment and the subsequent activation of tumor antigen-specific CD^8+^ T lymphocytes. Consequently, we hypothesize that the durable PFS achieved with immuno-chemotherapy in our patient may be, at least in part, attributable to prior crizotinib exposure.

## Implication and limitation

This study presents the first reported ES-SCLC case with ROS1 fusion, along with the different interventions employed. Initial attempts at targeted therapy with crizotinib led to primary drug resistance within 3 weeks. In contrast, a subsequent second-line treatment combining immunotherapy with chemotherapy, followed by immunotherapy maintenance, yielded durable drug responses (mPFS >19 months). These findings suggest that crizotinib may not confer clinical benefits for ES-SCLC patients with ROS1 fusions, whereas immuno-chemotherapy may prove to be a more effective therapeutic strategy for this patient cohort. Currently, there is a notable paucity of clinical evidence concerning ROS1 fusions in SCLC, and this study contributes preliminary insights into this underexplored area within the clinical landscape.

Several limitations of this study warrant consideration. Due to financial constraints, the patient declined repeat genomic profiling following disease progression, which precluded a thorough evaluation of potential resistance mechanisms and consequently limited evidence-based selection of subsequent therapies. This represents a significant limitation of the study. Additionally, It is based on a single-patient case report, rendering its conclusions provisional and serving only as a preliminary reference for managing ES-SCLC patients with ROS1 fusion. further investigation is necessary to determine whether ROS1 functions as a driver oncogene in SCLC. The specific efficacy and mechanism of response to crizotinib and immuno-chemotherapy in patients with ROS1 fusion remain unclear. Consequently, additional high-quality preclinical and clinical studies are essential to substantiate these issues.

## Conclusion

This research provides an important contribution by documenting the first case of an ES-SCLC patient with a ROS1 fusion, accompanied by TP53, RB1, PTEN, and TERT mutations. Following the administration of crizotinib as first-line treatment, the patient exhibited primary resistance; thus, the treatment was escalated to a second-line regimen involving chemotherapy combined with ICI, followed by ICI maintenance therapy. This approach resulted in a PR and a PFS significantly superior to the previously reported efficacy of immuno-chemotherapy in ES-SCLC patients (19 months compared to 5.7 months), underscoring the potential of immuno-chemotherapy in this setting. Based on the clinical evidence presented, we suggest that ROS1 may not act as a driver oncogene in ES-SCLC. Additionally, the presence of multiple mutations, various ROS1 fusion partners, and distinct ROS1 breakpoints in this case could influence the responsiveness to crizotinib therapy. The combination of ICI with chemotherapy and subsequent ICI maintenance therapy may provide enhanced therapeutic efficacy for patients with ES-SCLC, potentially due to the existence of ROS1 fusions alongside TP53 and TERT mutations. Given the complex genomic landscape and high heterogeneity characteristic of SCLC, further clinical investigations are imperative to explore and validate these findings.

## Data Availability

The original contributions presented in the study are included in the article/Supplementary Material, further inquiries can be directed to the corresponding authors.
