# Supplementary figures and images for: Case report: Durable response of immuno-chemotherapy targeting a rare ROS1 fusion-positive extensive-stage SCLC patient after primary resistance to crizotinib
Source: Front Pharmacol. 2025 Apr 29;16:1522542. doi: 10.3389/fphar.2025.1522542 (PMC12069334; doi:10.3389/fphar.2025.1522542)

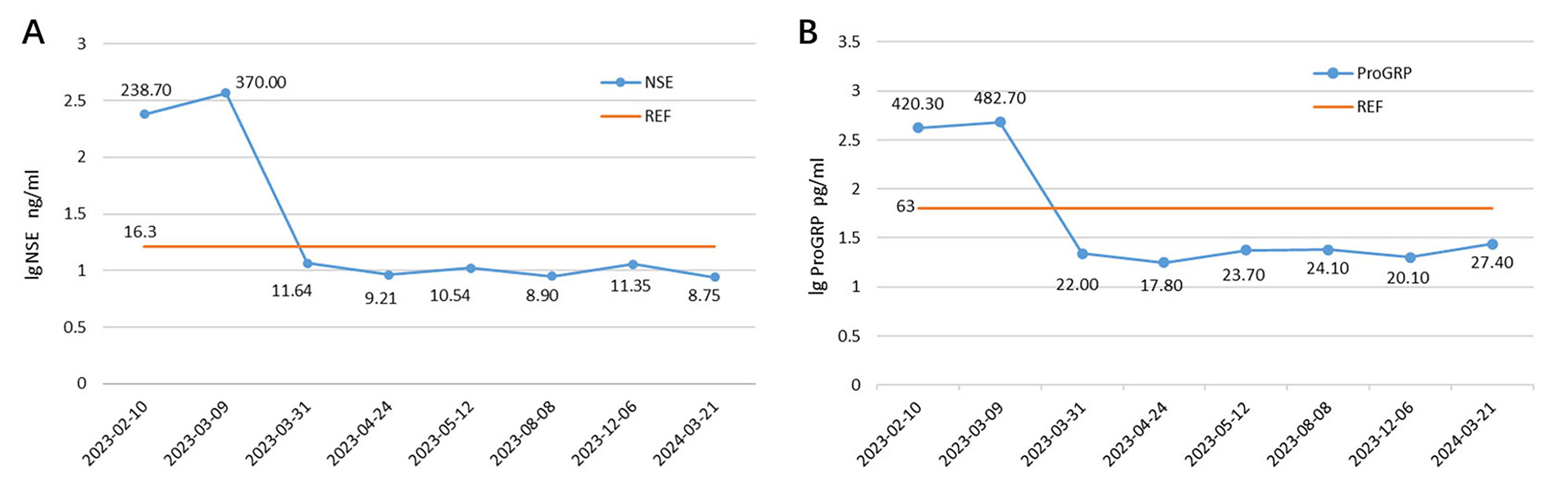

Supplement: Supplementary file 2 [file Image1.jpeg]
